# Endosymbiont diversity across native and invasive brown widow spider populations

**DOI:** 10.1038/s41598-024-58723-2

**Published:** 2024-04-12

**Authors:** Monica A. Mowery, Laura C. Rosenwald, Eric Chapman, Yael Lubin, Michal Segoli, Thembile Khoza, Robin Lyle, Jennifer A. White

**Affiliations:** 1 Mitrani Department of Desert Ecology, Blaustein Institutes for Desert Research, Ben-Gurion University of the Negev, Sede Boqer Campus, Midreshet Ben-Gurion, Israel; 2 Department of Entomology, University of Kentucky, Lexington, KY USA; 3 South African National Biodiversity Institute, Biosystematics Division, Pretoria, South Africa; 4 Agricultural Research Council-Plant Health and Protection, Biosystematics Division, Queenswood, South Africa; 5 Present Address: Department of Biology, York College, The City University of New York, Jamaica, NY USA

**Keywords:** Evolution, Microbiology

## Abstract

The invasive brown widow spider, *Latrodectus geometricus* (Araneae: Theridiidae), has spread in multiple locations around the world and, along with it, brought associated organisms such as endosymbionts. We investigated endosymbiont diversity and prevalence across putative native and invasive populations of this spider, predicting lower endosymbiont diversity across the invasive range compared to the native range. First, we characterized the microbial community in the putative native (South Africa) and invasive (Israel and the United States) ranges via high throughput 16S sequencing of 103 adult females. All specimens were dominated by reads from only 1–3 amplicon sequence variants (ASV), and most individuals were infected with an apparently uniform strain of *Rhabdochlamydia*. We also found *Rhabdochlamydia* in spider eggs, indicating that it is a maternally-inherited endosymbiont. Relatively few other ASV were detected, but included two variant *Rhabdochlamydia* strains and several *Wolbachia*, *Spiroplasma* and Enterobacteriaceae strains. We then diagnostically screened 118 adult female spiders from native and invasive populations specifically for *Rhabdochlamydia* and *Wolbachia.* We found *Rhabdochlamydia* in 86% of individuals and represented in all populations, which suggests that it is a consistent and potentially important associate of *L. geometricus. Wolbachia* was found at lower overall prevalence (14%) and was represented in all countries, but not all populations. In addition, we found evidence for geographic variation in endosymbiont prevalence: spiders from Israel were more likely to carry *Rhabdochlamydia* than those from the US and South Africa, and *Wolbachia* was geographically clustered in both Israel and South Africa. Characterizing endosymbiont prevalence and diversity is a first step in understanding their function inside the host and may shed light on the process of spread and population variability in cosmopolitan invasive species.

## Introduction

When moving into new habitats, invasive species may bring along microbial associates that can influence the invasion process^1,2^. Some microbes are vertically inherited endosymbionts that are restricted to the invasive species, but may yet influence interactions between the invasive and native species. Maternally-inherited endosymbionts have been shown to affect traits important to host fitness such as dispersal^3^, fecundity^4^, and defenses against natural enemies^5^, potentially providing an advantage to the invasive species^6^. Some endosymbionts affect the organism’s reproductive biology, for example, by modifying offspring sex ratio in infected populations, which can affect the speed of invasive spread^7^. For example, acting as both a mutualist and reproductive manipulator, *Rickettsia* caused whiteflies to have higher fitness and a higher proportion of daughters, and quickly spread in invasive populations^8^.

Assessing endosymbiont prevalence across geographically distant populations can provide a key to understanding the role of a symbiont. Widespread prevalence of a facultative endosymbiont suggests that the symbiont plays a functional role in its host, such as providing fitness benefits or manipulating reproduction^9,10^. The latter is often manifested by sex ratio distortions, although the most common reproductive manipulation is cytoplasmic incompatibility (CI), which causes incompatibilities between infected males and uninfected females but does not alter the sex ratio of the population^11^. Our understanding of the dynamics and prevalence of facultative endosymbiont infection during invasive spread is limited, especially for non-insect arthropod endosymbionts.

Invasive populations are predicted to exhibit reduced endosymbiont prevalence and diversity compared to native populations. During founding events, often few individuals are initially introduced into the invasive range^12^, in which case only a subset of endosymbionts found in the native range might be introduced to the new location^13^. However, in most biological invasions, multiple introductions are common^14^, and so endosymbiont diversity might be lower initially, and then increase over time as more individuals arrive from various localities^15^. Comparing endosymbiont diversity across invasive and native populations can provide valuable insights into the gain and loss of microbial communities during the invasion process.

The brown widow spider, *Latrodectus geometricus* (Theridiidae), is a medically important spider with neurotoxic venom. *Latrodectus geometricus* has spread recently to multiple locations around the world from the putative native range in southern Africa, most likely via cargo shipments^16,17^. Evidence suggests that during invasion, establishment and spread, spider traits related to dispersal, fecundity, and body size shifted across populations that were established over different time periods^18^. In addition to these shifts in ecologically important traits, associations with other organisms, such as parasitoids^19^ or endosymbionts, may have also changed during the invasion spread.

Endosymbionts of widow spiders (genus *Latrodectus*) are poorly known. A previous study on *L. geometricus* identified the endosymbiont *Rhabdochlamydia,* but only examined a few adult females in a single, inbred lab population in Florida, USA^20^. The same study did not detect *Rhabdochlamydia* in two other *Latrodectus* species. Hence, a further study across field-collected individuals worldwide is necessary to assess the presence of *Rhabdochlamydia* more broadly across populations of *L. geometricus*. The family Rhabdochlamydiaceae (Phylum: Chlamydiota) is predicted to be the most diverse chlamydial family^21^. It includes important vertebrate and human pathogens and is widespread across soil and aquatic ecosystems with many yet unknown hosts^22^. The genus *Rhabdochlamydia* has been found in a few distantly-related invertebrate hosts*,* including a cockroach^23^, a tick^24^, a dwarf spider^25^, and a terrestrial isopod^26^, although it was not found at a high prevalence within any of these species.

Also previously found in invasive populations of *L. geometricus* was *Wolbachia,* as a facultative associate in varying prevalence across populations^27^. *Wolbachia* infection is common in arthropods, with 40–60% of species infected^28^, as well as in other invertebrates including nematodes^29^. *Wolbachia* is known to affect the fitness and reproduction of many of its hosts, which could have implications for successful invasive establishment and spread^30^.

In this study, we compared endosymbiont presence and diversity across populations of the brown widow spider, *L. geometricus,* from the putative native range in South Africa to populations in the invasive range in the United States and Israel, using both high-throughput sequencing and diagnostic PCR screens. Our objectives were to (1) characterize the dominant endosymbionts in *L. geometricus*, (2) compare prevalence and diversity across purported native and known invasive ranges, and (3) investigate geographic patterns of endosymbiont infection within countries. We predicted that, due to founder effects, some endosymbionts would be lost and infection rates would be lower in invasive populations in the U.S. and Israel compared to putative native populations in South Africa, and that geographic patterns of endosymbiont loss would reflect the proposed routes of invasive spread of *L. geometricus* within each country*.*

## Methods

### Study species

*Latrodectus geometricus,* the brown widow spider, is a globally invasive species that has established populations in parts of North and South America, the Middle East, Australia, and Asia^17^. In the United States, *L. geometricus* was first detected in Miami, Florida in 1936^31^, was confined to southern Florida until the late 1990s, and was subsequently detected in Texas and California in the 2000s^32^. In Israel, *L. geometricus* was first detected in the Tel Aviv area in 1980^33^, and in the Negev region after 2000^34^. Throughout the global invasive range, *L. geometricus* is found in urban and settled habitats, and builds nests on and around buildings, on fences, garden furniture, trash bins, and in playgrounds^17^.

### Study sites

We collected *L. geometricus* adult females from urban environments across the United States (Edisto Island, South Carolina *n* = 10; Gainesville, Florida *n* = 10; Austin, Texas *n* = 6; Los Angeles, California *n* = 7), Israel (Haifa *n* = 7, Tel Aviv *n* = 10, Be’er Sheva *n* = 10, Yeruham *n* = 8, Midreshet Ben-Gurion *n* = 10, Eilat *n* = 1), and South Africa (Modimolle *n* = 10, Pretoria *n* = 5, Johannesburg *n* = 5, Kimberley *n* = 8, Cape Town *n* = 5, Riebeeck-Kasteel *n* = 6, George *n* = 7). Spiders were deprived of food for one week before they were preserved in 100% ethanol. Starved individuals have minimal gut content and are less likely to result in false positives for endosymbionts found in the spider’s prey^35^. To learn about the potential for vertical transmission, we also collected *L. geometricus* egg sacs from two sites in South Africa: Kimberley (*n* = 1) and Riebeeck Kasteel (*n* = 2), and sampled egg sacs produced in the laboratory from Midreshet Ben-Gurion (*n* = 3) and Tel Aviv (*n* = 2), Israel.

### Bacterial 16S sequencing

We surface-sterilized each adult female *L. geometricus* specimen (*n* = 125) with a series of bleach and ethanol rinses^36^ before longitudinally dividing the abdomen in half and extracting DNA from one half using DNeasy Blood and Tissue extraction kits (Qiagen, Germantown, MD) according to manufacturer’s instructions. In addition, we extracted DNA from the legs of two specimens, as well as from the eggs of 8 *L. geometricus* egg sacs to assess endosymbiont presence outside reproductive tissues and the potential for maternal transmission, respectively. Extraction quality for each sample was verified by PCR amplification of a ~ 650 bp segment of the COI gene (forward primer, lco1490: 5′-GGTCAACAAATCATAAAGATATTGG-3′, reverse primer, hco2198: 5′-TAAACTTCAGGGTGACCAAAAAATCA-3′; cycling conditions: one cycle of 94 °C for 3 min, followed by 35 cycles of 95 °C for 30 s, 53 °C for 30 s, 72 °C for 1 min, final extension at 72 °C for 5 min^37^. If COI failed to amplify, we attempted a second extraction with the other half of the abdomen. If this extraction failed to amplify product as well, we assumed sample preservation had been poor and eliminated the specimen from the dataset entirely (7/125 specimens).

To investigate which endosymbionts were present in these specimens, we profiled the microbiomes using high-throughput sequencing of the bacterial community. We amplified the V4 region of bacterial 16S rRNA for each sample using dual indexed 515F/806R primers^38^. We visualized the resulting products, and multiplexed 1 µl aliquots from successful amplifications into one of two libraries that were purified with GenCatch PCR Cleanup Kits. Samples that failed to amplify (6/118 samples) were not included in the library. Each library also included specimens from other projects that are not reported here, and received a PhiX spike to increase sequence heterogeneity among the amplified sequences. Libraries were sequenced at the University of Kentucky genomics core facility on an Illumina Miseq instrument using a paired-end strategy and 250 bp reads. Sequences from each run were demultiplexed, trimmed and quality filtered within BaseSpace (Illumina, basespace.illumina.com), then imported into QIIME2 (v2021.11, https://qiime2.org^39^) using a manifest. We conducted additional quality control using deblur^40^ implemented in QIIME2 using default parameters and a trim length of 251 bases. Resulting amplicon sequence variants (ASV) were taxonomically classified using a naïve Bayes classifier that was trained on the 515F/806R V4 region of the Greengenes 13_8 99% OTUs reference database^41^. We filtered out 15 ASV that originated from other specimens in the sequencing run (e.g., obligate endosymbionts of other host taxa, see^42^ for discussion of index swapping), which collectively constituted only a small minority (0.14%) of the 3.57 × 10^6^ reads associated with the *L. geometricus* samples. Following filtering, *L. geometricus* samples with less than 1000 reads were excluded from further analysis (9/112 adult samples, 6/8 egg samples). For the remaining samples, we blasted high prevalence ASV sequences (> 1% of any *L. geometricus* sample) against the NCBI nt database using the megablast algorithm, to identify bacterial taxa that may not have been included in the reference database. For ASV that appeared at very high prevalence or frequency (> 90% of reads for any specimen, or found in multiple specimens across multiple locations), we amplified a longer segment of 16S using universal primers from specimen(s) dominated by that taxon, to aid in taxonomic placement (Forward primer, 27F: 5′-AGAGTTTGATCMTGGCTCAG-3′, reverse primer 1492R: 5′-GGTTACCTTGTTACGACTT-3′, cycling conditions: one cycle of 95 °C for 2 min, followed by 35 cycles of 92 °C for 30 s, 55 °C for 30 s, 72 °C for 30 s, final extension at 72 °C for 6 min^43^.

### Diagnostic PCR

We diagnostically screened all samples (all 118 adult female, 8 egg, and 2 leg samples) for the two bacterial genera previously identified from *L. geometricus*: *Wolbachia* (Class Alphaproteobacteria, Order Rickettsiales, Family Anaplasmataceae) and *Rhabdochlamydia* (Class Chlamydiia, Order Chlamydiales, Family Rhabdochlamydiaceae^20,27^). For *Wolbachia*, we followed previously published protocols^44^, using primers specific to the *Wolbachia* surface protein (*wsp*) gene (Forward primer, wspF1: 5′-GTCCAATARSTGATGARGAAAC-3′, reverse primer wspR1: 5′-CYGCACCAAYAGYRCTRTAAA-3′, cycling conditions: one cycle of 94 °C for 2 min, followed by 36 cycles of 94 °C for 30 s, 59 °C for 45 s, 72 °C for 1 min 30 s, final extension at 70 °C for 10 min^44^. For *Rhabdochlamydia*, we designed new primers in Primer3^45^ to amplify a ~ 540 bp segment of 16S: Rhabdo_108F 5′-ACACTGCCCAAACTCCTACG-3′ and Rhabdo_647R 5′-TTAGCTWCGACACAGCCAGG-3′. All reactions were run in 10 µl volume; the *Rhabdochlamydia* reactions included 3 μl purified water, 1 μl 10× Buffer (New England Biolabs), 1.2 μl 10 mM dNTPs, 1.5 μl 25 mM MgCl_2_, 0.6 μl each of forward and reverse primers at 5 μM, and 0.1 μl of 5 U New England Biolabs Taq Polymerase. PCR reactions received one cycle of 94 °C for 2 min, followed by 25 cycles of 95 °C for 15 s, 56 °C at 15 s, 68 °C for 45 s. We electrophoresed and visualized the products on 1% agarose gels stained with Gel Red (Biotium) alongside known positive and negative (reagents-only) controls. Samples with initial negative diagnoses were retested before being categorized as uninfected. For a subset of the samples with positive evidence of infection, we repeated the PCR at a 20 µl volume and purified the PCR product with either GenCatch PCR Cleanup or Gel Extraction Kits (Epoch Life Sciences, Missouri City, TX) according to manufacturer’s instructions. Products were then submitted for Sanger sequencing (Eurofins, Louisville, KY). Resulting sequences were compared to the NCBI nucleotide database using the megablast algorithm, and specimens returning a 97% or higher match to the expected bacterial genus were scored as positive. For each strain of *Wolbachia*, we sequenced 5 MLST genes (*coxA*, *fbpA*, *ftsZ*, *gatB* and *hcpA*) and the *Wolbachia* surface protein (*wsp*) according to Baldo et al.^44^.

For *Rhabdochlamydia,* we ran phylogenetic analyses to place the *L. geometricus* strains, using a set of accessions across Chlamydiia with *Oligosphaera ethanolica* as an outgroup. For each analysis, multiple alignments were assembled using the MAFFT server (v. 7; https://mafft.cbrc.jp/alignment/server/^46^) using the Q-INS-I alignment method that takes secondary structure into account. Maximum likelihood phylogenetic analyses were conducted on 1576-character aligned datasets using Garli (v. 2.01^47^). We applied the most complex model available (GTR + I + G^48^) as per recommendations of Huelsenbock and Rannala^49^ for likelihood-based analyses. We conducted a 100-replicate ML search for the tree of highest log-likelihood and a 500-replicate ML bootstrap analysis^50^ with two search replicates per individual bootstrap replicate. All analyses used the default settings.

We used the same approach to generate a *Wolbachia* phylogeny. We used a concatenated data set containing 5 MLST genes (*coxA*, *fbpA*, *ftsZ*, *gatB* and *hcpA*; total of 2079 characters) with 38 *Wolbachia* strains pulled from the Wolbachia PubMLST website (https://pubmlst.org/organisms/wolbachia-spp^51^). Because rooting *Wolbachia* trees is challenging^52^, and our objective was only placement of our new strains within established *Wolbachia* supergroups, we chose to simply root the tree within Supergroup A.

Individual specimens were scored for the presence of *Rhabdochlamydia* and *Wolbachia* based on the combination of diagnostic, high-throughput, and Sanger sequencing data. For a sample to be scored positive, a positive diagnostic PCR needed to be corroborated by either high-throughput or Sanger sequencing validation. For a sample to be scored negative, consistent negative diagnostic PCRs needed to be accompanied by positive validation of spider COI and/or other bacterial taxa.

### Statistical methods

All analyses were conducted in R version 4.0.2^53^. To compare the prevalence of the dominant strains of *Rhabdochlamydia* and *Wolbachia* across South Africa, Israel, and the United States, we used a general linear model (“lme4” package^54^) with a binomial link function, with *Rhabdochlamydia1* or *Wolbachia1* presence or absence in an individual as the response variable, and country as the predictor. Maps showing collection localities in South Africa, Israel, and the United States were generated using the R package ggspatial^55^.

## Results

**Figure 1:**
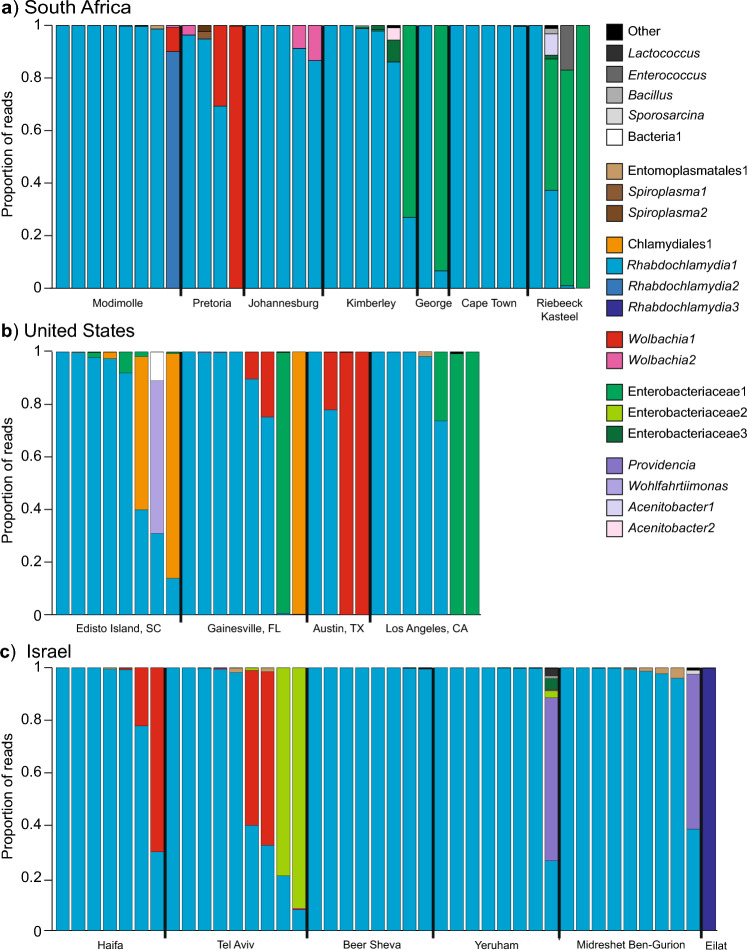
High throughput analysis of bacterial associates in *Latrodectus geometricus*. Proportional distribution of 16S sequencing reads from *L. geometricus* adult females collected from South Africa (**a**), the United States (**b**), and Israel (**c**). All bacterial strain types that exceeded 1% of reads in any sample are depicted. All remaining strains are collected within the “other” category. See Supplementary table 1 for taxonomic identification and raw read numbers for all amplicon sequence variants.

Compared to most microbiomes in arthropods, *L. geometricus* spiders have a depauperate microbial fauna. Of 103 adult female spiders that produced sufficient read depth (mean ± SE of 33,844 ± 2026 sequences per sample), all were dominated by one to three bacterial strains that accounted for greater than 90% of the reads (Fig. 1). In 64 samples, a single strain accounted for greater than 99% of reads. In most samples, the most prevalent bacterial ASV was *Rhabdochlamydia* (83/103 samples) although a few samples each were dominated by ASVs corresponding to *Wolbachia* (6 samples), Enterobacteriaceae (10 samples), *Providencia* (2 samples), *Wohlfahrtimonas* (1 sample) and a bacteria that could not be placed by the Greengenes reference database, but which our analyses (see below) place within the Chlamydiales (Chlamydiales1, 3 samples, Fig. 1).

Most samples had at least some *Rhabdochlamydia* representation. Nine samples from several locations in South Africa and the United States had negligible representation (< 0.1% of reads) of *Rhabdochlamydia*. The number of *Rhabdochlamydia* reads in the latter samples ranged from 0 (out of 4222 reads) to 359 (out of 37,618 reads), and most fell below the number of *Rhabdochlamydia* reads seen in blanks (9–81 reads). Two samples were diagnostically positive for *Rhabdochlamydia* despite low numbers of reads, and were additionally validated by Sanger sequencing of the diagnostic product, thus were counted as *Rhabdochlamydia* positive in the final dataset. In the remaining seven specimens, the low number of proportional reads and the diagnostic absence supports the genuine absence of *Rhabdochlamydia*. Of the additional 15 samples that were excluded from high throughput analysis due to poor initial amplification or insufficient read depth, six were validated to have *Rhabdochlamydia* and nine did not.

**Figure 2:**
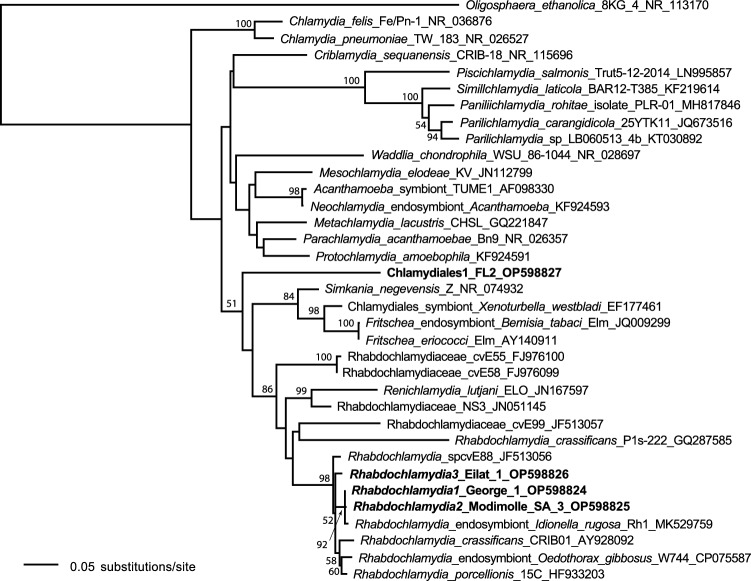
Phylogenetic placement of Chlamydial bacterial associates of *Latrodectus geometricus.* Tree of highest log likelihood from 500 maximum likelihood searches of a 35 OTU 16S data set containing 1576 characters conducted with Garli (v. 2.01) using the default settings. Taxa in bold are the new strains from *L. geometricus* (labeled Rhabdochlamydia1, 2, 3 and Chlamydiales1). Numbers above the nodes are bootstrap values above 50 (500 bootstrap replicates with 2 searches per replicate).

To gain insight into the occurrence of strains of the major endosymbionts found, we used Sanger sequencing data to distinguish among strains of the same symbiont clade. Most detected *Rhabdochlamydia* strains were identical (GenBank Accession #OP598824). Two variant strains were detected, each in one individual. The variant strain from a Modimolle, South Africa specimen (#OP598825) was 99.8% similar to the dominant strain, differing at only 1/480 bases of 16S. The variant strain from Eilat, Israel (#OP598826) was 98.8% similar, differing at 6/480 bases of 16S. Phylogenetically, all three strains were clustered together within the genus *Rhabdochlamydia* and family Rhabdochlamydiaceae (Fig. 2).

*Wolbachia* was much less common than *Rhabdochlamydia*, found in 14% (17/118) of individuals, but represented in spiders collected from all three regions. We were able to sequence all MLST genes and *wsp* for all three strains of *Wolbachia* (accession numbers OP612314-OP612330), except *gatB* in *L. geometricus Wolbachia3*. The most widespread and characteristic strain of *Wolbachia* in *L. geometricus*, *Wolbachia1*, was present in 13/118 specimens (11%), and phylogenetic analysis placed the strain in *Wolbachia* Supergroup F (Supplementary Fig. 1). In contrast*, L. geometricus Wolbachia2*, which was found in four specimens across three localities in South Africa, belongs to a different *Wolbachia* clade, Supergroup B. A third *Wolbachia* strain, *L. geometricus Wolbachia3,* which was found in a single sample that had not been included in high throughput sequencing but was validated with diagnostic PCR and subsequent sequencing, was placed in Supergroup A.

Only 16 other ASV, besides *Rhabdochlamydia* and *Wolbachia,* were ever found at > 1% prevalence in any sample, and the majority of these (nine) were each found in single specimens. Enterobacteriaceae1 represented a substantial proportion of reads in 12 individuals across several locations in South Africa and the United States, and was the dominant ASV in eight individuals. When blasted against the NCBI database, a 1359bp segment of 16S from this bacterium (#OP598828) was not closely aligned to any other accessions, bearing greatest resemblance to aphid secondary symbionts (e.g., EU348326 at 96.8%) or *Gilliamella*, a specialized honeybee gut symbiont (e.g., CP048265 at 95.84%). Enterobacteriaceae1 was absent from Israel, although a different Enterobacteriaceae ASV was detected from two individuals collected from one location in Israel. Two other gammaproteobacteria ASVs, *Providencia* and *Wohlfahrtiimonas*, were present in two and one specimens, respectively. One bacterial strain, which was found in four individuals across two locations in the southeast U.S., was not able to be placed against the Greengenes database in the QIIME2 pipeline, but a 498 bp segment of 16S aligns most closely with other Chlamydiales in GenBank (e.g. FJ976094 at 87.2%). Our chlamydial phylogeny (Fig. 2), also supports placement within this order, hence we have designated it Chlamydiales1. Other bacterial ASV were only found at a low percentage of reads across spiders (two *Acinetobacter* ASV*,* two *Spiroplasma* ASV*,* and one each of *Entomoplasmatales, Sporosarcina, Bacillus, Enterococcus,* and *Lactococcus*)*.*

Comparing across the three countries, a higher proportion of spiders collected in Israel were infected with the dominant strain of *Rhabdochlamydia, Rhabdochlamydia1,* than spiders from South Africa (GLM, *z* = − 2.128, *p* = 0.033) or the U.S. (GLM, *z* = − 2.538, *p* = 0.011)*.* We found no differences in prevalence of the dominant *Wolbachia* strain, *Wolbachia1,* across countries (GLM, US-Israel, *z* = − 0.689, *p* = 0.491; US-South Africa, *z* = − 1.268, *p* = 0.205; Israel-South Africa, *z* = − 0.669, *p* = 0.504)*.* Using diagnostic PCR screening, we found evidence for *Rhabdochlamydia* in 100% (8/8) of *L. geometricus* eggs tested from South Africa and Israel. In contrast, only two out of eight egg sacs showed signal of *Wolbachia,* both from Tel Aviv*,* consistent with the proportional infection rate in adults from the source populations*.*

**Figure 3:**
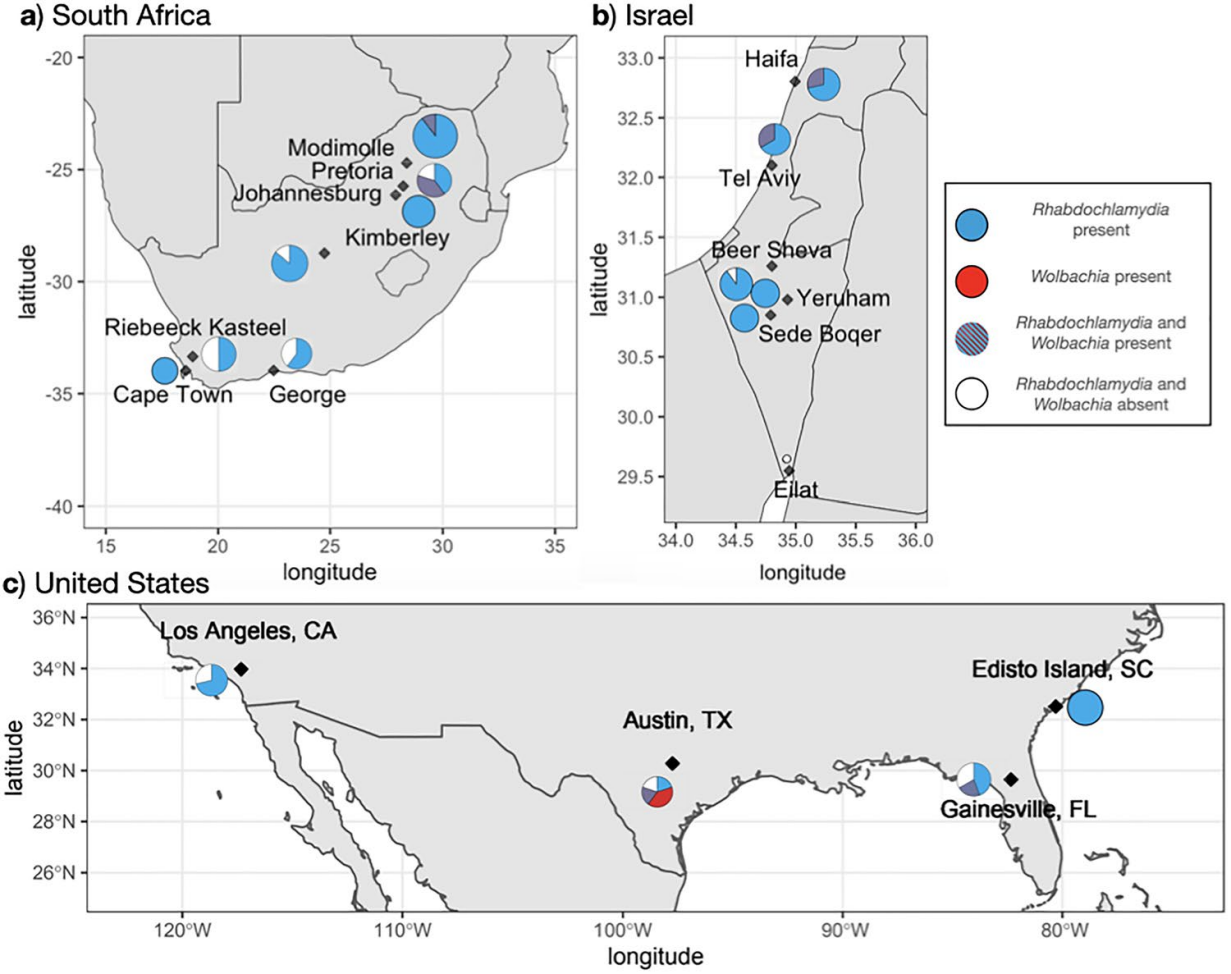
Proportion of adult female *L. geometricus* infected with *Rhabdochlamydia1* and/or *Wolbachia1* detected through PCR screening across 17 localities in (**a**) South Africa, (**b**) Israel, and (**c**) the United States. Blue represents individuals infected with just *Rhabdochlamydia1,* purple represents individuals infected with both *Rhabdochlamydia1* and *Wolbachia1,* red represents individuals infected with just *Wolbachia1,* and white represents individuals infected with neither *Wolbachia1* nor *Rhabdochlamydia1.* Size of pie charts corresponds to the number of individual spiders screened from each site (range = one specimen from Eilat, Israel to 10 specimens from Edisto Island, SC, USA, see Supplementary table 1 for sample sizes and collection localities).

*Wolbachia* prevalence was too low for formal spatial analysis, but visually appeared to have some level of clustering (Fig. 3). In South Africa, both *Wolbachia1* and *Wolbachia2* were found in northeastern populations (Johannesburg, Pretoria, and Modimolle) but were not detected elsewhere in the country. Likewise, in Israel, *Wolbachia1* was present in central and northern populations (Tel Aviv and Haifa), but was not detected in the southern Negev populations (Beer Sheva, Yeruham, Sede Boqer, Eilat). Among the four U.S. populations, *Wolbachia1* was found in spiders collected from Florida and Texas, *Wolbachia3* was in South Carolina, but no *Wolbachia* was detected in spiders from California, the most recently detected invasive population.

## Discussion

*Latrodectus geometricus* spiders have maintained a characteristic microbiome throughout their global spread. We identified one predominant endosymbiont, *Rhabdochlamydia1* in almost all spiders (86%), and represented in all collection locations. We also found a characteristic Supergroup F *Wolbachia* (*Wolbachia1*) represented in all countries, albeit in fewer individuals (11% of spiders). We detected both *Rhabdochlamydia1* and *Wolbachia1* in *L. geometricus* eggs, indicating that both are vertically transmitted endosymbionts.

The widespread presence of *Rhabdochlamydia* suggests that it might be important functionally for the host. In other arthropods, endosymbionts found at consistently high frequency across wide geographic ranges have often subsequently been found to have important fitness or reproductive consequences for their hosts^56,57^. Little is known about the functional role of *Rhabdochlamydia* in arthropods*.* It was described from a variety of mostly non-insect arthropods and was generally found at low prevalence in the tested populations^23,24,26^. In a terrestrial isopod, *Rhabdochlamydia* had pathogenic effects^26^. The high prevalence (86%) and vertical transmission of *Rhabdochlamydia* in *L. geometricus* argue against a strongly pathogenic role for this bacterial strain within our system. Genomic analysis of *Rhabdochlamydia* found in other arthropod hosts, an isopod and a tick, found pathways for polyamine synthesis^22^, which are relevant for virulence and stress responses, suggesting that some strains of this bacteria are potentially beneficial in their host.

We also detected *Rhabdochlamydia* in *L. geometricus* legs, consistent with the work of Dunaj et al.^20^, which indicated that the bacteria is found throughout the body and not just restricted to reproductive tissue. Dunaj et al.^20^ also found that the bacterial community of *L. geometricus* was dominated by *Rhabdochlamydia*, lacking the microbial diversity of the other spider species they examined, and speculated that this result may have been an artifact of laboratory-reared, inbred *L. geometricus* spiders. Our field collected spiders from locations around the world suggest that their result was not an artifact, but a genuine representation of a characteristic and depauperate bacterial community in *L. geometricus*. Vertically transmitted bacterial symbionts often dominate the sampled microbiomes of their hosts, overwhelming the signal from more casual bacterial associates^11,25,35^.

Importantly, maternal transmission of *Rhabdochlamydia* suggests the possibility of reproductive manipulation of host by symbiont. Reproductive manipulation is extremely common in vertically transmitted symbionts, and the list of bacteria that have been demonstrated to induce such manipulations is rapidly expanding^11,58^. *Rhabdochlamydia* has not yet been tested for host reproductive manipulation. The widespread prevalence and vertical transmission of *Rhabdochlamydia* in *L. geometricus* would make this system an excellent prospect for such investigations.

*Latrodectus geometricus* was host to several strains of *Wolbachia*, a bacterial clade well known for reproductive manipulation. *Wolbachia* is common in spiders, but most strains belong to Supergroup A or B, as is the case in insects^30^. In contrast, the dominant *Wolbachia* strain in *L. geometricus* belongs to Supergroup F, which has rarely been reported for spiders. Supergroup F has been found sporadically in arthropods, including South African scorpions^59^, termites^60^, quill mites^61^, and nematodes^62^. Preliminary work on *L. geometricus* suggested that *Wolbachia* might induce mild CI in this species^63^, but the strain of *Wolbachia* was not characterized, and additional experiments will be necessary to fully validate CI in this system.

Although symbiont communities were largely similar across our sampled regions, we did find some subtle differences between the likely native and invasive ranges. *Rhabdochlamydia* was found at highest prevalence in Israel compared to populations in the U.S. and South Africa. Multiple strains of *Rhabdochlamydia, Wolbachia,* and the Enterobacteriaceae were found in South Africa, the putative native population*.* The dominant strain of Enterobacteriaceae was found in South Africa and the U.S., but absent in Israel, the newest invasive region that we sampled. From a previous study*, Wolbachia* prevalence in *L. geometricus* in the U.S. was highest near the initial site of introduction in Florida^27^. In comparison, we found lower *Wolbachia* prevalence in other locations in the southeastern and central U.S, and absence in spiders from California, the most recently established population. Similarly, in Israel, *Wolbachia* was absent in recently established populations in southern Israel. These patterns are consistent with the loss of endosymbionts during the invasion process, but more localities, specimens, and more knowledge of the invasion route is needed. Climatic differences such as hotter, dryer conditions in the Negev Desert in southern Israel could also contribute to reduction of *Wolbachia*^64^, although deeper sampling effort would be needed to assess whether *Wolbachia* is entirely absent from these locations.

Further work will test the functional role and fitness effects of endosymbiont presence in *L. geometricus*, as well as compare patterns of host-endosymbiont diversity during invasive spread. Invasive *L. geometricus* are highly dispersive^18^, and are less susceptible to parasitism by parasitoids compared to native widow species in the invasive range^19^. It would be valuable to test whether these advantages and others during invasion are related to interactions with endosymbionts. In particular, the dominance and high prevalence of *Rhabdochlamydia* across global populations of *L. geometricus* suggests an important role of this endosymbiont. Characterizing potentially important and widespread endosymbionts is a step towards understanding their relevance to ecological interactions and responses to rapid environmental changes.

### Supplementary Information

Supplementary Information.

Supplementary Figure 1.

## Data Availability

The datasets generated and/or analyzed during the current study are available via NCBI SRA, Bioproject PRJNA1068539: https://www.ncbi.nlm.nih.gov/bioproject/?term=PRJNA1068539.
